# Risk of Neuropathy with Celiac Disease

**DOI:** 10.15844/pedneurbriefs-29-7-6

**Published:** 2015-07

**Authors:** J. Gordon Millichap

**Affiliations:** 1 Division of Neurology, Ann & Robert H. Lurie Children's Hospital of Chicago, Chicago, IL; 2 Departments of Pediatrics and Neurology, Northwestern University Feinberg School of Medicine, Chicago, IL

**Keywords:** Neuropathy, Celiac Disease, Electroencephalogram

## Abstract

Investigators from Columbia University College of Physicians and Surgeons, New York, Karolinska Institutet, Stockholm, and Orebro University, Sweden, examined the risk of developing neuropathy in a nationwide population-based sample of 28,232 patients with biopsy-verified celiac disease (CD).

Investigators from Columbia University College of Physicians and Surgeons, New York, Karolinska Institutet, Stockholm, and Orebro University, Sweden, examined the risk of developing neuropathy in a nationwide population-based sample of 28,232 patients with biopsy-verified celiac disease (CD). Data were obtained from small-intestinal biopsies performed at Sweden's 28 pathology departments between June 1969 and February 2008. Risk of neuropathy in CD patients was compared with that of 139,473 age and sex-matched controls. Most study participants were females (61.7%), and 11,763 (41.7%) patients with CD were diagnosed in childhood. Median age of diagnosis was 29 years (range 0-95 years). Patients were followed for a median of 10 years. During follow-up, 198 patients with CD and later diagnosis of neuropathy (0.7%) were identified vs 359 controls (0.3%) with later diagnosis of neuropathy. CD was associated with a 2.5-fold increased risk of later neuropathy (P<.001). Also, risk was increased for chronic inflammatory demyelinating neuropathy (P=.001), autonomic neuropathy (P=.009), and mononeuritis multiplex (P=.006), but not for acute inflammatory demyelinating polyneuropathy (P=.68). Adjusting for educational level, diabetes, autoimmune thyroid disease, and vitamin deficiencies had only a marginal effect on risk estimate. Age and gender did not influence the risk of neuropathy in patients with CD, but risk was highest in the first year after diagnosis of CD. The increased risk for neuropathy was observed both before and after diagnosis of CD. CD screening is recommended in patients with neuropathy. [1]

COMMENTARY. Celiac disease, a common genetically-based food intolerance, may appear at any age and may be manifested by extra-intestinal as well as gastrointestinal symptoms. Diagnosis requires a high degree of suspicion, a screening test of serum autoantibody anti-tissue transglutaminase, confirmation by intestinal biopsy, and response to treatment with a strict gluten-free diet [2]. Neurological disorders that affect 10% of CD patients include ataxia, neuropathy, vestibular dysfunction, migraine and seizures. The epilepsy prevalence is 1% to 5%, and a gluten-free diet sometimes controls seizures with CD that are refractory to AEDs.

Investigators in Gaziantep, Turkey, have determined the frequency of epileptiform discharges in the EEGs of 307 children with CD and 197 age- and sex-matched controls. Of 25 patients with epileptiform discharges (spike/sharp wave discharges) 24 (7.8%) were in the CD group and 1 (0.5%) was in the control group. (P=0.001). Among the 24 with CD, 21 (9.7%) were in the newly diagnosed –untreated CD group and 3 (3.3%) were in a gluten-free diet group (P=0.03). Patients diagnosed with CD and having symptoms suggestive of seizures should be evaluated with an EEG; those with epileptiform discharges should follow strict adherence to a gluten-free diet that may decrease the risk of seizures [3].
